# Natural catalytic immunoglobulins hydrolyzing histones as a link between inflammation and humoral immunity in schizophrenia

**DOI:** 10.1192/j.eurpsy.2021.1451

**Published:** 2021-08-13

**Authors:** E. Ermakov, V. Buneva, G. Nevinsky

**Affiliations:** 1 Laboratory Of Repair Enzymes, Institute of Chemical Biology and Fundamental Medicine, Novosibirsk, Russian Federation; 2 Department Of Natural Sciences, Novosibirsk State University, Novosibirsk, Russian Federation

**Keywords:** schizophrénia, Inflammation, Histones, Humoral immunity

## Abstract

**Introduction:**

Schizophrenia pathogenesis is known to be associated with chronic low-grade inflammation. Inflammation can be caused by extracellular histones that are released from cells due to apoptosis dysfunction. It can also be accompanied by the formation of natural catalytic immunoglobulins that bind and hydrolyze histones.

**Objectives:**

To investigate the ability to hydrolyze various histones by polyclonal IgGs from serum of patients with schizophrenia.

**Methods:**

We recruited 50 patients (28 men and 22 women) with a verified diagnosis of paranoid or simple schizophrenia and 25 healthy individuals (13 men and 12 women) in our study. IgG preparations were obtained by affinity chromatography and analyzed by SDS-PAGE and MALDI MS. Catalytic activity of IgGs were revealed by the degree of hydrolysis of five histones using SDS-PAGE. To prove that antibodies exhibit histone-hydrolyzing activity, we used rigorous generally accepted criteria. Statistical analysis was performed in Origin 2019.

**Results:**

IgGs of patients are shown to bind and hydrolyze various histones with high efficiencies. The IgGs histone-hydrolyzing activity level, depending on the type of histone (H1, H2a, H2b, H3, H4), was statistically significantly 6–20 times higher than that of healthy individuals (Fig. 1). However, only 21% of patients with schizophrenia had IgGs with very high activity. The IgGs activity level correlated with PANSS General scale.

**Figure:**
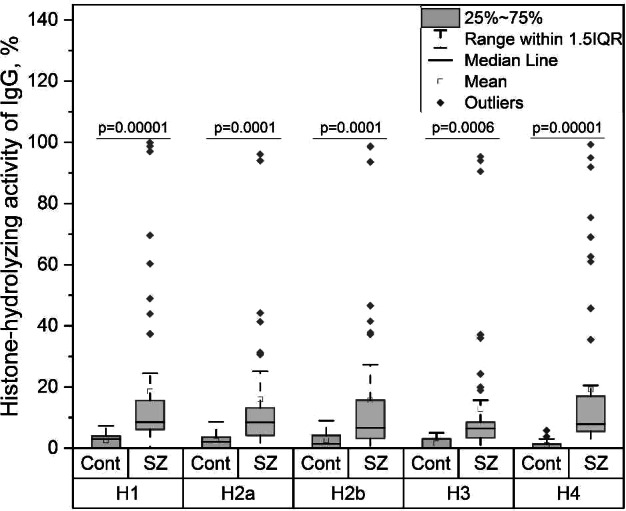

Fig.1. Histone-hydrolyzing activity of IgG.

**Conclusions:**

We suggest that histone-hydrolyzing antibodies may play a compensatory role in schizophrenia because removal of extracellular histones minimizes the inflammatory responses. Therefore, such IgGs may be the link between inflammation and humoral immunity, and also be a promising biomarker.

**Conflict of interest:**

This work was supported by Russian Foundation for Basic Research under grant 20-015-00156. E.A.E. is the recipient of the fellowship of the President of the Russian Federation (SP-2258.2019.4).

